# The *Drosophila *homolog of the mammalian imprint regulator, CTCF, maintains the maternal genomic imprint in *Drosophila melanogaster*

**DOI:** 10.1186/1741-7007-8-105

**Published:** 2010-07-30

**Authors:** William A MacDonald, Debashish Menon, Nicholas J Bartlett, G Elizabeth Sperry, Vanya Rasheva, Victoria Meller, Vett K Lloyd

**Affiliations:** 1 Department of Biology, Dalhousie University, Halifax, Nova Scotia, Canada; 2 Department of Biological Sciences, Wayne State University, Detroit, MI, USA; 3 Department of Biology, Mt. Allison University, Sackville, New Brunswick, Canada; 4 Instituto de Tecnologia Quimica e Biologica, Universidade Nova de Lisboa, Oeiras, Portugal

## Abstract

**Background:**

CTCF is a versatile zinc finger DNA-binding protein that functions as a highly conserved epigenetic transcriptional regulator. CTCF is known to act as a chromosomal insulator, bind promoter regions, and facilitate long-range chromatin interactions. In mammals, CTCF is active in the regulatory regions of some genes that exhibit genomic imprinting, acting as insulator on only one parental allele to facilitate parent-specific expression. In *Drosophila*, CTCF acts as a chromatin insulator and is thought to be actively involved in the global organization of the genome.

**Results:**

To determine whether CTCF regulates imprinting in *Drosophila*, we generated *CTCF *mutant alleles and assayed gene expression from the imprinted *Dp(1;f)LJ9 *mini-X chromosome in the presence of reduced *CTCF *expression. We observed disruption of the maternal imprint when *CTCF *levels were reduced, but no effect was observed on the paternal imprint. The effect was restricted to maintenance of the imprint and was specific for the *Dp(1;f)LJ9 *mini-X chromosome.

**Conclusions:**

CTCF in *Drosophila *functions in maintaining parent-specific expression from an imprinted domain as it does in mammals. We propose that *Drosophila *CTCF maintains an insulator boundary on the maternal X chromosome, shielding genes from the imprint-induced silencing that occurs on the paternally inherited X chromosome.

See commentary: http://www.biomedcentral.com/1741-7007/8/104

## Background

The correct establishment and propagation of epigenetic states are essential for normal development, and disruption of these processes leads to disease. Genomic imprinting is a striking example of the effect of epigenetics on gene regulation. In genomic imprinting, a mark, the imprint, is imposed on the two parental genomes during gametogenesis. In the zygote, the imprint is maintained through each mitotic division and results in the parental alleles of a gene, or entire homologous chromosomes, adopting different epigenetic states. As a result of these different epigenetic states, one parental allele can be silenced while the allele from the other parent, although identical in DNA sequence, is active.

The CCCTC-binding factor, CTCF, is a key player in maintaining epigenetically distinct chromatin domains. CTCF is an evolutionarily conserved zinc finger-containing DNA-binding protein that can function both directly in gene regulation as a transcription factor and also indirectly by mediating long-range chromatin interactions. In this latter role, CTCF acts as a chromatin insulator by isolating enhancer and promoter regulatory units and as a barrier to the spread of heterochromatin [1]. CTCF binds at multiple sites throughout the genome [2-4], indicating a widespread role in generating chromatin domains. Epigenetic isolation is necessary for correct maintenance of genomic imprints as imprinted domains are often interspersed among nonimprinted domains [5,6], necessitating their isolation from flanking regulatory regions. Additionally, the two homologous alleles must be isolated as differential gene expression patterns, chromatin conformations, and replication timing have all been associated with imprinted alleles [7].

CTCF binding has been reported at multiple mammalian imprinted domains [5], illustrating the importance of insulator function in maintaining parent-specific expression. The role of CTCF in imprinting has been best characterized for the mammalian *Igf2/H19 *genes, in which only the maternal *H19 *allele and paternal *Igf2 *alleles are expressed [8-10]. On the maternal chromosome, CTCF binds to a differentially methylated domain (DMD) located between the *Igf2 *and *H19 *genes, preventing interaction of downstream enhancer sequences with the promoter of *Igf2*, effectively silencing the gene. Methylation of the paternal DMD effectively blocks CTCF binding, allowing activation of *Igf2 *expression while also initiating the silencing of the *H19 *gene. Binding of CTCF is necessary to maintain the epigenetic state of the imprinted alleles. Consequently, if the CTCF binding site in the *Igf2/H19 *DMD is mutated, the monoallelic expression arising from the imprint is lost [11,12]. An additional facet of CTCF binding appears to be the facilitation of higher-order chromatin structures through DNA looping, a property which fortifies the silencing of *Igf2 *and the activation of *H19 *on the maternal chromosome [13,14]. The details of CTCF binding and its consequences are less well studied at other imprinted loci; however, its insulator function and role in establishing higher-order chromatin function appear to be shared features of other mammalian imprinted loci which bind CTCF [5,15-17]. The KvDMR1 imprinted domain, which contains two CTCF binding sites, regulates the tissue-specific expression of the gene *Cdkn1c*. It has been suggested that the tissue-specific imprinting of *Cdkn1c *is due to tissue-specific binding of CTCF to the KvDMR1 imprint domain [18,19]. The imprinted domain *Wsb1/Nf1 *also requires CTCF-mediated interchromosomal association with the *Igf2/H19 *imprinted domain for proper parent-specific expression [20].

Although CTCF appears to be the major insulator protein in vertebrates, the more compact *Drosophila *genome uses a variety of insulator proteins, among which is the *Drosophila *CTCF homolog dCTCF [21-23]. The insulator activity of dCTCF has been well characterized in the bithorax complex, where it demarcates the chromatin domains that define separate regulatory regions [22,24,25], and, as in mammals, dCTCF is widely used as an insulator throughout the *Drosophila *genome and also acts directly as a transcription factor [26-28]. Though the role of CTCF in the formation of distinct chromatin domains is conserved from *Drosophila *to mammals, the roles of CTCF in epigenetic processes such as genomic imprinting have been assumed to differ [1]. To assess the effect of dCTCF on *Drosophila *imprinting, we used a well-characterized imprinting assay system, the *Dp(1;f)LJ9 *mini-X chromosome, in which a readily visible eye color gene, *garnet *(*g*), is juxtaposed to an imprint control region and so becomes a marker for imprinting [29]. Regulation of the *Dp(1;f)LJ9 *imprint previously has been shown to share properties of mammalian imprinting, including transcriptional silencing of gene clusters and differential chromatin states between homologues [30-32]. Here we present the first demonstration that *dCTCF *has a role in the regulation of genomic imprinting in *Drosophila*. As is the case in mammalian imprinting, *dCTCF *in *Drosophila *is involved in the regulation of the maternal imprint by maintaining parent-specific expression from the maternally inherited X chromosome.

## Results

### Characterization of *CTCF *alleles

The *CTCF*^*EY15833 *^allele (FBrf0132177) was produced by insertion of the *P{EPgy2} *element into the +26 position relative to the transcription start site of the *dCTCF *gene by the Berkeley Drosophila Genome Project (BDGP) Gene Disruption Project [33]. Homozygous *CTCF*^*EY15833 *^adults appear healthy and are reasonably fertile. *CTCF*^*30*^, created by a partial deletion of the *P{EPgy2} *element, is homozygous lethal. *CTCF*^*30 *^lacks the entire 5' end of the *P{EPgy2} *element but retains 4860 bp of the 3' end. Flanking *dCTCF *sequences and the *quemao *(*qm*) gene remain intact in *CTCF*^*30*^. The reduced severity of *CTCF*^*EY15833*^, with an intact *P{EPgy2} *element, suggests that a promoter in the 5' end of *P{EPgy2} *may partially rescue *dCTCF *expression. To test this idea, we measured the *dCTCF *transcript levels by quantitative real-time PCR (qRT-PCR) of third instar larvae. Expression in homozygous *CTCF*^*EY15833 *^larvae is 20% ± 4% of that in wild-type (*y w*) controls. This is consistent with previous studies showing that *CTCF*^*EY15833 *^homozygotes produce ~50% of wild-type dCTCF protein levels [22]. Homozygous *CTCF*^*30 *^larvae cannot be recovered in sufficient numbers for qRT-PCR, but heterozygous *CTCF*^*30*^/+ larvae display 68 ± 9% of wild-type transcript levels, consistent with a severe reduction in expression by this mutation. Taken together, the phenotypic and expression analysis of *dCTCF *alleles indicates that both *CTCF*^*EY15833 *^and *CTCF*^*30 *^alleles have reduced *dCTCF *expression and that the 5' end of *P{EPgy2} *may drive sufficient expression to allow recovery of *CTCF*^*EY15833 *^adults.

### *Drosophila *CTCF maintains the maternal imprint of the *garnet *gene on the *Dp(1;f)LJ9 *mini-X chromosome

**Figure 1:**
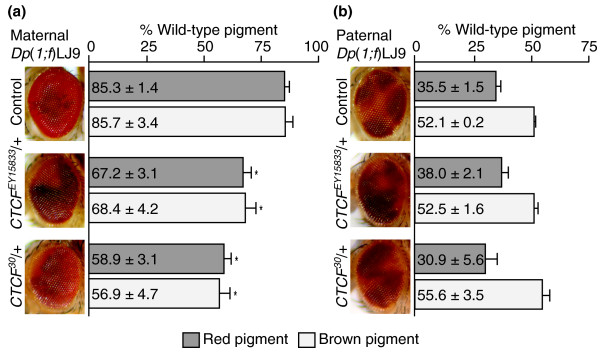
**Effect of *dCTCF *alleles on the imprinted *Dp(1;f)LJ9 garnet *(*g*) gene expression**. **(a) **Maternally transmitted *Dp(1;f)LJ9 *mini-X chromosome; the control (*y*^1^*z*^*a*^*g*^*53d*^/*Dp(1;f)LJ9*) displays full *garnet *expression with no variegation observed. Both *dCTCF *mutant alleles tested (*y*^1^*z*^*a*^*g*^*53d*^/*Dp(1;f)LJ9*; *CTCF*^*EY15833*^/+ and *y*^1^*z*^*a*^*g*^*53d*^/*Dp(1;f)LJ9*; *CTCF*^*30*^/+) disrupt maintenance of the maternal imprint, causing variegated *garnet *gene expression. Significant reduction in both red and brown pigment levels is observed in the presence of *CTCF*^*EY15833*^ or *CTCF*^*30 *^alleles. **(b) **Paternally transmitted *Dp(1;f)LJ9 *mini-X chromosome; the control (*y*^1^*z*^*a*^*g*^*53d*^/*Dp(1;f)LJ9*) exhibits variegated *garnet *gene expression, whereas the introduction of *CTCF*^*EY15833*^ and *CTCF*^*30 *^alleles had no significant effect on *garnet *gene variegation. Pigment assay values are expressed as a percentage of wild-type pigment levels ± standard deviation. Values that are significantly different from the controls are marked with an asterisk signifying *P *< 0.001.

To test the effect of the *dCTCF *alleles on *Drosophila *imprinting, we used the *Dp(1;f)LJ9 *mini-X chromosome. Maternal inheritance of the *Dp(1;f)LJ9 *mini-X chromosomes (*Dp(1;f)LJ9*^MAT^) generate full expression of the marker gene *garnet*, whereas paternal inheritance (*Dp(1;f)LJ9*^PAT^) generates variegated *garnet *expression (Figures 1a and 1b, control). The variegated *garnet *gene phenotype arising from paternal transmission is mitotically stable and so results in distinct clonal regions exhibiting *garnet *expression in an eye devoid of *garnet *expression. Expression of *garnet *affects both red (pteridine) and brown (ommochrome) eye pigments, which makes this mini-X chromosome an easily assayed system in which to assess the effect of *dCTCF *alleles on imprinting in *Drosophila*.

Transmission of the *Dp(1;f)LJ9 *mini-X chromosome through the female results in *y*^*1*^*z*^*a*^*g*^*53d*^/*Dp(1;f)LJ9*; +/+ mini-X chromosome-bearing male progeny with essentially wild-type expression of the *garnet *imprint marker gene. Eyes are phenotypically wild type, with 85.3 ± 1.4% wild-type red pigment levels and 85.7 ± 3.4% wild-type brown pigment levels (Figure 1a, control). To determine the effects of *dCTCF *on the maternal maintenance of imprinted *garnet *expression, the *CTCF*^*X *^alleles (*X *represents *CTCF*^*30 *^or *CTCF*^*EY15833*^) were crossed to females with the *Dp(1;f)LJ9 *mini-X chromosome: *y*^*1*^*z*^*a*^*g*^*53d*^/*Y*; *CTCF*^*X*^/*TM3, Sb Ser *males x X^X/*Dp(1;f)LJ9 *females. This cross-generated progeny with a mutant *dCTCF *allele and a maternally imprinted mini-X chromosome (*y*^*1*^*z*^*a*^*g*^*53d*^/*Dp(1;f)LJ9*^*MAT*^; *CTCF*^*X*^/+), which were compared with progeny similarly carrying a maternally imprinted chromosome, but wild type for *dCTCF*.

**Figure 2:**
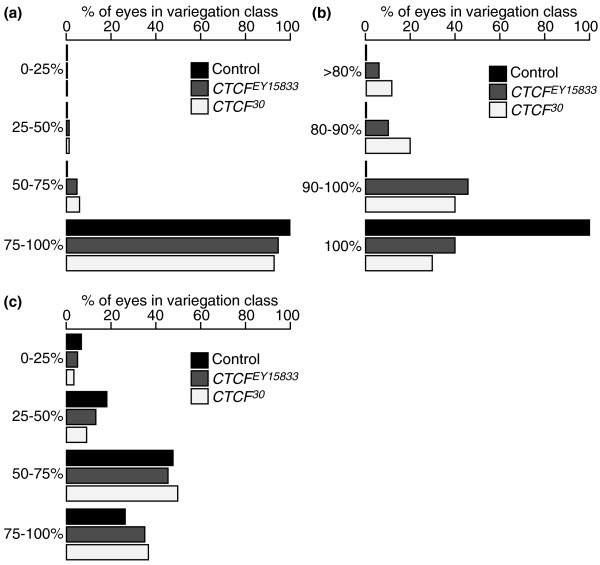
**Eye phenotype of *Dp(1;f)LJ9 Drosophila *with *dCTCF *alleles**. **(a) **Phenotypes of maternally inherited *Dp(1;f)LJ9 *mini-X chromosome ranging from 0% to 100% pigmentation; control *n *= 300, *CTCF*^*EY15833 *^*n *= 300, *CTCF*^*30 *^*n *= 300. **(b) **Phenotypes of maternally inherited *Dp(1;f)LJ9 *mini-X chromosome ranging from >80% to 100% pigmentation; control *n *= 150, *CTCF*^*EY15833 *^*n *= 132 (Kolmogorov-Smirnov test; *P *< 0.001) and *CTCF*^*30 *^*n*= 122 (Kolmogorov-Smirnov test; *P *< 0.001). **(c) **Phenotypes of paternally inherited *Dp(1;f)LJ9 *mini-X chromosome ranging from 0% to 100% pigmentation; control *n *= 300, *CTCF*^*EY15833 *^*n *= 300, *CTCF*^*30 *^*n *= 300. Each eye was scored depending on its phenotypic class, and the prevalence of each phenotypic class is expressed as a percentage versus the total number of eyes scored (*n*).

For each *dCTCF *allele tested, the mutant allele substantially reduced expression of the maternally transmitted imprint marker gene (Figure 1a). Progeny with a maternally inherited mini-X chromosome (*Dp(1;f)LJ9*^*MAT*^) with *CTCF*^*EY15833 *^reduced pigment levels to 67.2 ± 3.1% (*P *< 0.001) and 68.4 ± 4.2% (*P *< 0.001) for red and brown pigments, respectively. *Dp(1;f)LJ9*^*MAT *^progeny coupled with *CTCF*^*30 *^resulted in an even greater reduction of pigment levels: 58.9 ± 3.1% (*P *< 0.001) and 56.9 ± 4.7% (*P *< 0.001) for red and brown pigments, respectively. No variegated *garnet *expression was observed in flies with *Dp(1;f)LJ9*^*MAT *^and wild type for *dCTCF *(Figures 2a and 2b). However, when *Dp(1;f)LJ9*^*MAT *^was inherited along with mutant *dCTCF *alleles, variegated *garnet *expression was observed (Figures 2a and 2b). These results demonstrate that the maintenance of the maternal imprint is highly sensitive to dCTCF dosage.

**Figure 3:**
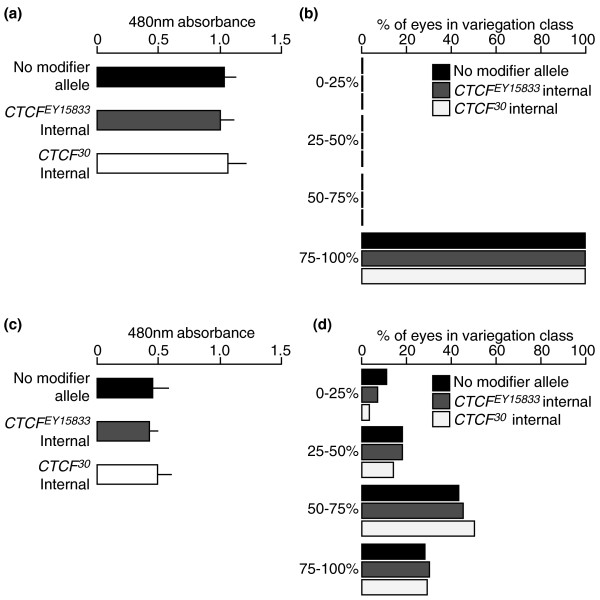
**Absence of maternal or paternal effects from mutant *dCTCF *on *Dp(1;f)LJ9 garnet *expression**. External control progeny (no modifier allele) have the same genotype as internal control progeny (*y*^*1*^*z*^*a*^*g*^*53d*^/*Dp(1;f)LJ9*; *TM3, Sb Ser*/+) but are generated from a separate cross with parents that have never encountered a mutant *CTCF*^*X *^allele. **(a) **Maternally inherited *Dp(1;f)LJ9 CTCF*^*X *^internal control eye pigment levels; no significant difference in pigment levels was observed between external control progeny (no modifier allele) and internal control progeny from fathers carrying *CTCF*^*X *^(*CTCF*^*X *^internal), demonstrating that no paternal effect occurs. **(b) **Phenotypes of maternally inherited *Dp(1;f)LJ9 *ranging from 0% to 100% pigmentation; No modifier allele *n *= 300, *CTCF*^*EY15833 *^internal *n *= 300, *CTCF*^*30 *^internal *n *= 300. No *garnet *variegation was detected from the internal controls. **(c) **Paternally inherited *Dp(1;f)LJ9 CTCF *internal control eye pigment levels; no significant difference in pigment levels was observed between the external control progeny (no modifier allele) and internal control progeny from mothers carrying *CTCF*^*X *^(*CTCF*^*X *^internal), demonstrating that no maternal effect occurs. **(d) **Phenotypes of paternally inherited *Dp(1;f)LJ9 *ranging from 0% to 100% pigmentation; no modifier allele *n *= 300, *CTCF*^*EY15833 *^internal *n *= 300, *CTCF*^*30 *^internal *n *= 300. No significant change in *garnet *variegation was detected from the internal controls. Red eye pigment levels are measured by absorbance at 480 nm, and pigment quantification mean values for each group are based on *n *= 5 samples (40 heads total); error bars represent standard deviation.

This cross also produced sibling progeny that have a maternally inherited *Dp(1;f)LJ9*^MAT^, but with the balancer chromosome, and so wild type for *dCTCF *(described in "Methods," genotype: *y*^*1*^*z*^*a*^*g*^*53d*^/*Dp(1;f)LJ9*^MAT^; *TM3, Sb Ser */+). These internal control flies are genotypically identical to the external controls but have the male parent mutant for *CTCF*^*X *^and so would allow detection of any paternal effect. None was detected (Figures 3a and 3b).

### *Drosophila *CTCF does not regulate the paternal imprint of the *Dp(1;f)LJ9 *mini-X chromosome

Variegated silencing of *garnet *from the paternally inherited *Dp(1;f)LJ9*^PAT ^is a consequence of the spreading of heterochromatin from the imprinted region [29,32]. In contrast to the effects observed when the *Dp(1;f)LJ9 *is maternally imprinted, *dCTCF *mutants had no effect on the paternal expression of the *garnet *imprint marker gene. Paternal inheritance of the mini-X chromosome (X^Y/*Dp(1;f)LJ9 *males crossed to *y*^*1*^*z*^*a*^*g*^*53d*^/*y*^*1*^*z*^*a*^*g*^*53d*^; *TM3, Sb Ser*/+ females) results in *y*^*1*^*z*^*a*^*g*^*53d*^/*Dp(1;f)LJ9*^*PAT*^; +/+ progeny with variegated *garnet *expression and a marked reduction in eye pigment levels (35.5 ± 1.5% red and 52.5 ± 0.2% brown wild-type pigment levels; Figure 1b, control). The introduction of *dCTCF *mutant alleles (*y*^*1*^*z*^*a*^*g*^*53d*^/*y*^*1*^*z*^*a*^*g*^*53d*^; *CTCF*^*X*^/*TM3, Sb Ser *females to X^Y/*Dp(1;f)LJ9 males*) to generate progeny with either mutant *CTCF*^*EY15833 *^or *CTCF*^*30 *^alleles and a paternally imprinted *Dp(1;f)LJ9*^*PAT *^mini-X chromosome (*y*^*1*^*z*^*a*^*g*^*53d*^/*Dp(1;f)LJ9*; *CTCF*^*X*^/+), yielded no significant change in either red or brown eye pigment levels or phenotype (Figures 1b and 2c).

Sibling progeny with a paternally inherited *Dp(1;f)LJ9*^PAT ^along with the balancer chromosome are wild type for *dCTCF *(described in "Methods," genotype: *y*^*1*^*z*^*a*^*g*^*53d*^/*Dp(1;f)LJ9*; *TM3, Sb Ser*/+) and can be used to determine whether there is a maternal effect from mothers mutant for *CTCF*^*X*^. No maternal effect was detected; progeny wild type for *dCTCF *from mothers with either *CTCF*^*EY15833 *^or *CTCF*^*30 *^showed no significant change in *garnet *expression levels (Figures 3c and 3d).

### *Drosophila *CTCF does not regulate the establishment of the maternal or paternal imprint of the *Dp(1;f)LJ9 *mini-X chromosome

**Figure 4:**
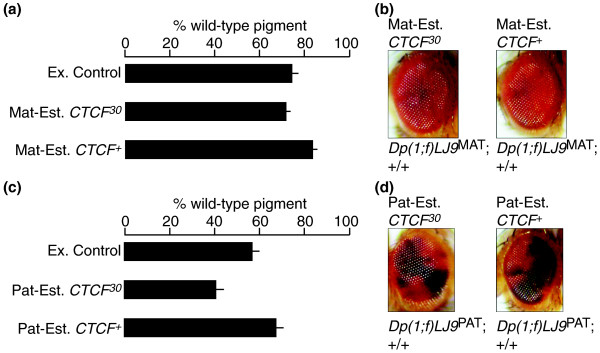
***dCTCF *does not affect establishment of the *Dp(1;f)LJ9 *imprint. All genotypes tested are *y*^*1*^*z*^*a*^*g*^*53d*^/*Dp(1;f)LJ9*; +/+ but differ in parental genotype**. External control progeny (Ex. Control) are generated from parents carrying *Dp(1;f)LJ9 *that have never been exposed to a mutant *dCTCF *allele. Progeny generated from a parent carrying both the imprinted *Dp(1;f)LJ9 *mini-X chromosome and a mutant *dCTCF *allele test for effects on imprint establishment (Mat or Pat-Est. *CTCF*^*30*^), whereas parental siblings carrying *Dp(1;f)LJ9 *and wild type for *CTCF *serve as an internal control (Mat or Pat-Est. *CTCF*^+^). **(a) **Maternal establishment of the *Dp(1;f)LJ9 *imprint is not affected by *CTCF*^*30*^. No significant change in red pigment levels was detected between external control progeny (Ex. Control; n = 23), mothers carrying *Dp(1;f)LJ9*^MAT^, and *CTCF*^*30 *^(Mat-Est. *CTCF*^*30*^; n = 10), and mothers carrying *Dp(1;f)LJ9*^MAT ^and *CTCF*^+^ (Mat-Est. *CTCF*^+^; n = 10). **(b) **No phenotypic difference is present between progeny generated from *Dp(1;f)LJ9 *carrying mothers, either mutant (Mat-Est. *CTCF*^*30*^) or wild type (Mat-Est. *CTCF*^+^), for *CTCF*. **(c) **Paternal establishment of the *Dp(1;f)LJ9 *imprint is not affected by *CTCF*^*30*^. No significant change in red pigment levels was detected between external control (Ex. Control; n = 44), fathers carrying *Dp(1;f)LJ9*^PAT ^and *CTCF*^*30 *^(Pat-Est. *CTCF*^*30*^; n = 18), and fathers carrying *Dp(1;f)LJ9*^PAT ^and *CTCF*^+ ^(Pat-Est. *CTCF*^+^; n = 29). **(d) **No phenotypic difference is present between progeny generated from *Dp(1;f)LJ9 *carrying fathers, either mutant (Pat-Est. *CTCF*^*30*^) or wild type (Pat-Est. *CTCF*^+^) for *dCTCF*.

To determine whether the effect of *dCTCF *was on the somatic maintenance of the imprint or its establishment in the germline of the parents, we examined the phenotype of progeny from male or female parents with both the *Dp(1;f)LJ9 *mini-X chromosome and a mutant *CTCF*^*30 *^allele. If *dCTCF *affects the establishment of the imprint, the imprint should be disrupted in the progeny of mutant *CTCF*^*30 *^parents, but not wild-type (*CTCF*^+^) parents. When we compared the phenotype of progeny wild type for *dCTCF *but differing in their parental genotype, no significant alternation in *garnet *expression levels resulted between *Dp(1;f)LJ9*^MAT ^progeny from mothers carrying *CTCF*^*30 *^(Figure 4a; Mat-Est. *CTCF*^*30*^) and either of the external or internal controls wild type for *dCTCF *(Figure 4a; Ex. Control and Mat-Est. *CTCF*^+^, respectively). This was reflected in the unchanged phenotype of progeny from mothers mutant or wild type for *dCTCF *(Figure 4b; Mat-Est. *CTCF*^*30 *^and Mat-Est. *CTCF*^+^, respectively). Likewise, mutant *dCTCF *did not effect the establishment of the paternal imprint. *Dp(1;f)LJ9*^PAT ^progeny from fathers carrying *Dp(1;f)LJ9 *and *CTCF*^*30 *^(Figure 4c; Pat-Est. *CTCF*^*30*^) had no significant change in *garnet *expression compared with either the external or internal controls (Figure 4c; Ex. Control and Pat-Est. *CTCF*^+^, respectively). Again, these *Dp(1;f)LJ9*^PAT ^progeny also had no observable change in phenotype between fathers mutant or wild type for *dCTCF *(Figure 4d; Pat-Est. *CTCF*^*30 *^and Pat-Est. *CTCF*^+^, respectively). These findings distinguish the function of dCTCF in the maintenance versus the establishment of the imprint on the *Dp(1;f)LJ9 *mini-X chromosome; dCTCF is involved in the maintenance of the imprint in the soma of progeny as its reduction disrupts the maternal imprint. However, dCTCF is not involved in the establishment of the imprint as the presence of mutant *CTCF*^*30 *^in either the maternal or paternal germline during establishment of the imprint does not affect regulation of the imprint.

### *Drosophila *CTCF is not a general modifier of position-effect variegation

**Figure 5:**
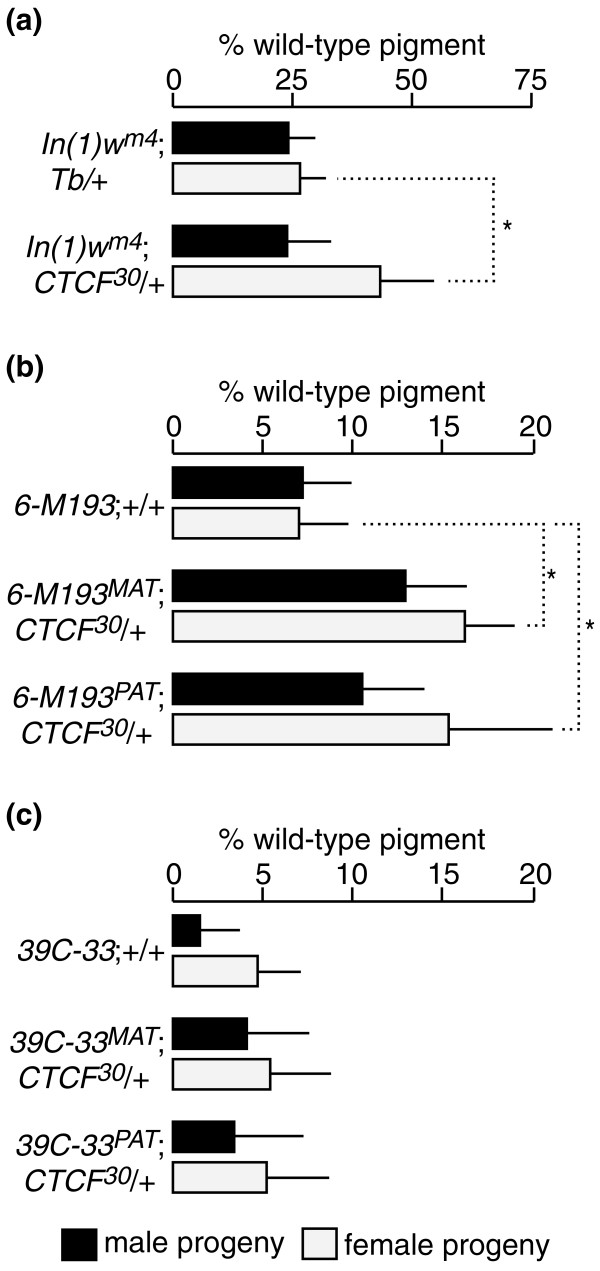
**The effect of *CTCF*^*30 *^on *white *variegation in *In(1)w*^*m4 *^and the fourth chromosome variegating strains *6-M193 *and *39C-33***. **(a) **Pigment levels were measured for *In(1)w*^*m4*^/*w*^1118^;*CTCF*^*30*^/*+ *and *In(1)w*^*m4*^/*Y*;*CTCF*^*30*^/*+ *genotypes compared with the corresponding sibling *In(1)w*^*m4*^;*Tb/+ *controls. *In(1)w*^*m4 *^heterozygous for *CTCF*^*30 *^results in an increase in *white *expression; however, this increase is only significant in female progeny (white bars). Red pigment quantification mean values for each group are based on *n *= 10 (50 heads total). Error bars represent standard deviation, and values significantly different from the controls are marked with an asterisk (*P *< 0.001). **(b) **Pigment levels were independently measured for both the maternally (*6-M193*^MAT^) and paternally (*6-M193*^PAT^) inherited fourth chromosome variegator *6-M193*. *6-M193 *heterozygous for *CTCF*^*30 *^results in an increase in *white *expression when either maternally or paternally inherited; however, this increase is only significant in female progeny (white bars). Red pigment quantification mean values for each group are based on *n *= 9 (45 heads total) for *6-M193*^MAT ^male and female progeny; *n *= 10 (50 heads total) for *6-M193*^PAT ^male and female progeny; *6-M193*;+/+ control male progeny; and *n *= 12 (60 heads total) for *6-M193*;+/+ control female progeny. Error bars represent standard deviation, and values significantly different from the controls are marked with an asterisk (*P *< 0.001). **(c) **Pigment levels were independently measured for both the maternally (*39C-33*^MAT^) and paternally (*39C-33*^PAT^) inherited fourth chromosome variegator *39C-33*. *39C-33 *heterozygous for *CTCF*^*30 *^results in no significant change in *white *expression when either maternally or paternally inherited. Red pigment quantification mean values for each group are based on *n *= 10 (50 heads total) for *39C-33*^MAT ^and *39C-33*^PAT ^male and female progeny, and *n *= 20 (100 heads total) for *39C-33*;+/+ control male and female progeny. Error bars represent standard deviation.

To determine the effect of *dCTCF *mutant alleles on the *Dp(1;f)LJ9*^MAT ^imprint, we tested the effect of *CTCF*^*30 *^on *In(1)w*^*m4*^, a classical variegating rearrangement [34], and two fourth chromosome transgenic constructs [35] in which the *white *(*w*) gene is variegated. Like the *Dp(1;f)LJ9 *mini-X chromosome, the variegated silencing in *In(1)w*^*m4 *^is induced by the centric heterochromatin of the X chromosome [35] and the fourth chromosome has been proposed to be evolutionarily related to the X chromosome [36]. We found that the *CTCF*^*30 *^allele decreased silencing of *white *in *In(1)w*^*m4*^;*CTCF*^*30*^/+ females while having no significant effect on *white *expression levels in *In(1)w*^*m4*^;*CTCF*^*30*^/+ males compared with sibling *In(1)w*^*m4*^;*Tb*/+ controls (Figure 5a). Similarly, the *6-M193 *strain responded to *CTCF*^*30 *^with a modest decrease in *white *reporter silencing in females only (Figure 5b), whereas the *39C-33 *strain showed no significant change in *white *reporter silencing from *CTCF*^*30 *^(Figure 5c). These results demonstrate that *CTCF*^*30 *^is not a ubiquitous modifier of variegated heterochromatic silencing in *Drosophila*, consistent with the absence of an effect on silencing of the paternally inherited *Dp(1;f)LJ9 *mini-X chromosome. Furthermore, the decreased silencing of the nonimprinted variegators is opposite to the effect of *CTCF*^*30 *^on *Dp(1;f)LJ9*^MAT ^silencing. Thus, the role of *dCTCF *in the maintenance of the maternal *Dp(1;f)LJ9 *imprint represents a distinct parent-specific function for *dCTCF *on the imprinted *Dp(1;f)LJ9 *mini-X chromosome.

## Discussion

CTCF is essential for insulator function in vertebrates, where it plays an active role in regulating imprinted gene expression. In *Drosophila*, dCTCF has likewise been shown to be involved in the insulator function of boundary elements [28,37]. Our results show that parent-specific expression from an imprinted domain in *Drosophila *is dependent on dCTCF function. Maintenance of expression from maternally inherited *Dp(1;f)LJ9 *mini-X chromosome is highly sensitive to dCTCF; even a modest decrease in *dCTCF *mRNA alters the maternal imprint so that it resembles the paternal imprint.

The effect of dCTCF on maternal-specific expression is limited to the maintenance of imprint. The presence of mutant *dCTCF *in either the maternal or paternal parents, when the imprint is being established, does not affect the imprint in the progeny. These results are strikingly similar to the role of CTCF in mammalian imprinting, where CTCF assists in the postfertilization formation of an imprinted region, but is dispensable for the establishment of an imprint [11,12,38].

Furthermore, the requirement for dCTCF for maintenance of the maternal *Dp(1;f)LJ9 *imprint is specific and does not represent a ubiquitous role for dCTCF in regulating heterochromatic silencing. Not only is the paternal *Dp(1;f)LJ9 *imprint unaffected by mutant *dCTCF*, but other variegating *Drosophila *reporter genes respond differently to mutant *dCTCF*. Thus, the association of *dCTCF *expression with the maintenance of the maternal *Dp(1;f)LJ9 *imprint boundary demonstrates a distinct function for *dCTCF *in imprinted gene expression.

In mammals, maternally imprinted regions that bind CTCF rely critically on this binding to insulate the imprinted loci and establish distinct chromatin domains. Our results show that a reduction in *dCTCF *levels disrupts the maternal imprint boundary on the *Drosophila Dp(1;f)LJ9 *mini-X chromosome, and consequently the marker gene, *garnet*, is silenced. Variegated silencing of *garnet *from *Dp(1;f)LJ9*^PAT ^inheritance is a consequence of heterochromatin formation, nucleated from the paternal imprint control region, spreading in *cis *[29,32]. The absence of an effect upon the introduction of *dCTCF *mutant alleles to *Dp(1;f)LJ9*^PAT ^suggests that dCTCF binding and boundary function occurs only on the maternal chromosome. Thus, it is conceivable that a reduction in *dCTCF *levels enables the spreading of heterochromatin on the maternal *Dp(1;f)LJ9*^MAT ^in a manner similar to that of the paternal *Dp(1;f)LJ9*^PAT^. This would suggest that dCTCF defines the boundary of a distinct maternal-specific imprinted chromatin domain required to maintain maternal-specific gene expression on the X chromosome.

The model organism Encyclopedia of DNA Elements (modENCODE) project provides detailed mapping of regulatory elements throughout the *Drosophila *genome [39]. Large-scale profiling of dCTCF insulator sites from early embryo modENCODE data reveals several candidate dCTCF insulator sites present proximal to the predicted heterochromatic breakpoint of the *Dp(1;f)LJ9 *mini-X chromosome. These dCTCF insulator sites, located between the centric heterochromatic imprinting center and the imprint marker gene *garnet*, could account for the sensitivity of the maternal imprint to dCTCF expression. If dCTCF were bound only when the X chromosome was transmitted maternally, mutations to *dCTCF *would disrupt insulator function and lead to maternal silencing of the imprint marker gene. Although such binding remains to be tested, it is similar to the function of CTCF at mammalian imprinted regions.

That the structure of CTCF and its role as an insulator, barrier, and transcriptional regulator is conserved between mammals and insects have been well established [23,27,28]. However, the finding that CTCF maintains its function in regulating the imprinting of diverse genes in such phylogenetically distinct organisms is remarkable. CTCF is a versatile DNA binding factor; subsets of its zinc fingers are adept at binding diverse DNA sequences, and the rest of the protein is able to maintain common regulator interactions and insulator function [40]. This feature may explain how CTCF can regulate imprinting in organisms as diverse as insects and mammals, in which the imprinted target sequences are different.

Previously, the evolutionary origin of imprinting has been extrapolated from the conservation of imprinting among specific genes. Such studies have led to the proposal that mammalian imprinting is of relatively recent origin and restricted to eutherian mammals [41,42]. However, studies showing that the molecular mechanism of imprinting is highly conserved have suggested a much more ancient origin [7,30,43]. Mammalian imprint control elements inserted into transgenic *Drosophila *act as discrete silencing elements [44,45] and can retain posttranscriptional silencing mechanisms involving noncoding RNA [46]. Whereas these transgenic imprinting elements lose their parent-specific functions, the retention of epigenetic silencing mechanisms suggests an ancient and conserved origin of imprinting mechanisms. Our finding that CTCF has a role in the maintenance of maternal imprints in insects, as it does in mammals, supports the possibility of evolutionary conservation for both CTCF function and the mechanisms of genomic imprinting.

## Conclusions

CTCF is a multifunctional protein with a conserved role as a chromosomal insulator in both mammals and *Drosophila*. To determine whether dCTCF is involved in imprinted regulation in *Drosophila *as it is in mammals, we generated a *dCTCF *mutant allele with severe reduction in *dCTCF *expression and tested its effects on the expression of the imprint marker gene, *garnet*, on the *Dp(1;f)LJ9 *mini-X chromosome. Full *garnet *gene expression, which occurs when the *Dp(1;f)LJ9 *mini-X chromosome is maternally inherited, was disrupted when *dCTCF *expression levels were reduced. No effect of reduced *dCTCF *expression was observed on the *Dp(1;f)LJ9 *mini-X chromosome when it was inherited paternally. The effect of *dCTCF *mutations is on the maintenance rather than on the establishment of the imprint, and is specific to the *Dp(1;f)LJ9 *mini-X chromosome. These results demonstrate that dCTCF is involved in maintaining parent-specific expression from the maternally inherited X chromosome in *Drosophila*, a role paralleling its involvement in mammalian imprinting.

## Methods

### *Drosophila *culture

All crosses were maintained at 22°C and cultured on standard cornmeal-molasses *Drosophila *media with methyl benzoate (0.15%) as a mold inhibitor. Each set of crosses was performed in 55-ml shell vials and contained 10-15 virgin females and 10-15 males. Each of the crosses was subcultured three or four times at 3-day intervals before the parents were discarded. Each cross was replicated four to six times, and the progeny were pooled. All stocks were obtained from the Bloomington *Drosophila *stock center, with the exception of the *CTCF*^*30 *^allele and the variegating fourth chromosome transgene strains. The *CTCF*^*EY15833 *^allele was created by insertion of P{EPgy2} 27 bp downstream of the *dCTCF *transcription start site. The homozygous lethal *CTCF*^*30 *^allele was generated by imprecise excision of *CTCF*^*EY15833*^. The *CTCF*^*30 *^deletion was characterized by amplification across the break and sequencing of the PCR product. *CTCF*^*30 *^is deleted for all 5' transposon sequences but retains 4860 bp at the 3' end. No genomic sequence was removed by the *CTCF*^*30 *^deletion. The fourth chromosome variegating strains were generated using the transposable P element *P [hsp26-pt, hsp70-w]*, which contains the *hsp70*-driven *white *gene that is susceptible to silencing caused by heterochromatin formation [47]. The *6-M193 *strain has the construct inserted within a *1360 *transposon and inside the *Syt7 *gene (fourth chromosome coordinate: 323400), whereas the *39C-33 *strain is generated from the construct being inserted into gene of the RNA binding protein *gawky *(fourth chromosome coordinate: 680211), which is in close proximity to a *1360 *transposon [47].

**Figure 6:**
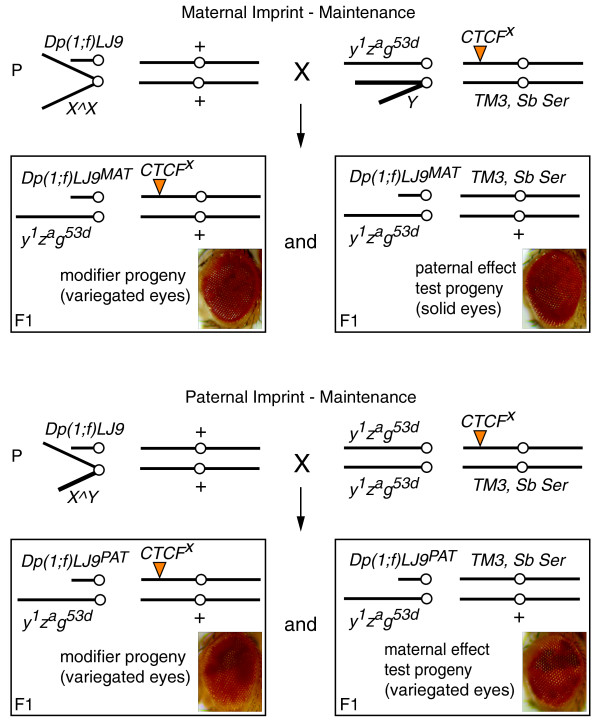
**Mating schematic for testing the effect of *dCTCF *on the maintenance of the *Dp(1;f)LJ9 *imprint**. Two sets of progeny are generated from this cross: progeny that have independently inherited the *Dp(1;f)LJ9 *mini-X chromosome and a *CTCF*^*X *^mutant allele (modifier progeny) and progeny that have inherited *Dp(1;f)LJ9 *and the *TM3, Sb Ser *balancer, but had a parent carrying a *CTCF*^*X *^mutant allele (maternal and paternal effect test progeny).

To determine the effect of mutant *dCTCF *on imprint maintenance, the *CTCF*^*30 *^and *CTCF*^*EY15833 *^alleles were crossed into *y*^*1*^*z*^*a*^*g*^*53d *^background to yield stable stocks of *y*^*1*^*z*^*a*^*g*^*53d*^/*y*^*1*^*z*^*a*^*g*^*53d*^; *CTCF*^*X*^/*TM3, Sb Ser *(where *CTCF*^*X*^ is the *CTCF*^*30 *^or *CTCF*^*EY15833 *^allele). To test the effect of a *dCTCF *allele on the paternal imprinting of *garnet*, *Dp(1;f)LJ9, y*^+^*g*^+^/*X^Y *males were crossed to *y*^*1*^*z*^*a*^*g*^*53d*^/*y*^*1*^*z*^*a*^*g*^*53d*^; *CTCF*^*X*^/*TM3, Sb Ser *females, and the reciprocal cross with *Dp(1;f)LJ9, y*^+^*g*^+^/*X^X *virgin females was performed to test the effect on the maternal imprinting of *garnet *(Figure 6). *y*^*1*^*z*^*a*^*g*^*53d*^/*Dp(1;f)LJ9*; *CTCF*^*X*^/*+ *male progeny were collected on the basis of wild-type *yellow *(*y*^+^) body color, which independently confirms the presence of the *Dp(1;f)LJ9 *chromosome, whereas the *zeste *allele (*z*^*a*^) reduces background eye color of the *g *allele (*g*^*53d*^). The *y*^*1*^*z*^*a*^*g*^*53d*^/*Dp(1;f)LJ9*; *TM3, Sb Ser*/*+ *sibling males were used as internal controls (Figure 6). The "no modifier" control test cross for paternal *garnet *imprinting consisted of *Dp(1;f)LJ9, y*^+^*g*^+^/*X^Y *males crossed to *y*^*1*^*z*^*a*^*g*^*53d*^/*y*^*1*^*z*^*a*^*g*^*53d*^; *TM3, Sb Ser/+ *females, with the reciprocal cross serving as the maternal control: *y*^*1*^*z*^*a*^*g*^*53d*^**/***Dp(1;f)LJ9*; *+*/*+ *and *y*^*1*^*z*^*a*^*g*^*53d*^/*Dp(1;f)LJ9*; *TM3, Sb Ser*/+ male progeny were collected as controls.

**Figure 7:**
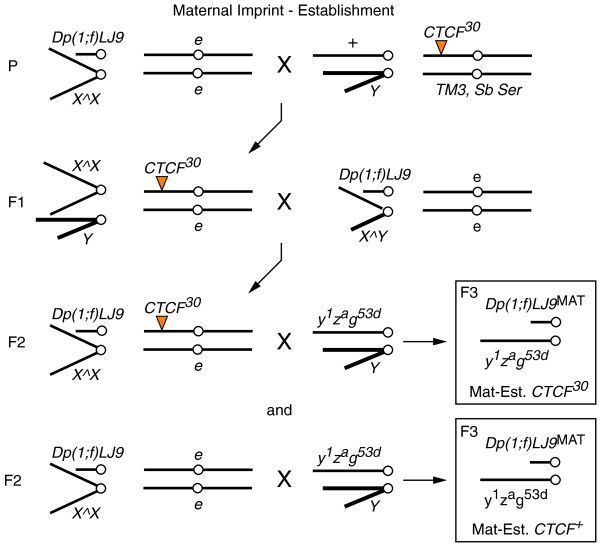
**Mating schematic for testing *dCTCF *for an effect on maternal establishment of the *Dp(1;f)LJ9 *imprint**. Two primary sets of progeny and an external control were generated from this cross: progeny with a maternally-inherited *Dp(1;f)LJ9 *mini-X chromosome from mothers with the *CTCF*^*30 *^mutation (Mat-Est. *CTCF*^*30*^) and progeny that also have a maternally imprinted *Dp(1;f)LJ9 *chromosome but from mothers wild type for *CTCF *(Mat-Est. *CTCF*^+^). External control crosses were produced by crossing F1 generation *X^X/Dp(1;f)LJ9; e/e *females to *y*^*1*^*z*^*a*^*g*^*53d*^/*Y *males (not depicted).

**Figure 8:**
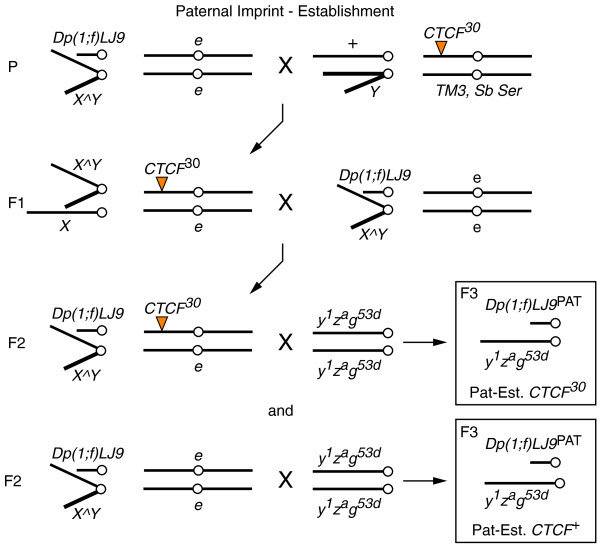
**Mating schematic for testing *dCTCF *for an effect on the paternal establishment of the *Dp(1;f)LJ9 *imprint**. Two primary sets of progeny and an external control were generated from this cross: progeny with a paternally-transmitted *Dp(1;f)LJ9 *mini-X chromosome from fathers with the *CTCF*^*30 *^mutation (Pat-Est. *CTCF*^*30*^) and progeny that also have a paternally imprinted *Dp(1;f)LJ9 *mini-X chromosome but from fathers wild type for *CTCF *(Pat-Est. *CTCF*^+^). External control crosses were produced by crossing F1 generation *X^Y/Dp(1;f)LJ9; e/e *males to *y*^*1*^*z*^*a*^*g*^*53d*^/*y*^*1*^*z*^*a*^*g*^*53d *^females (not depicted).

To test for the effects of *CTCF *on germline imprint establishment, mutant *CTCF *must be present in parents carrying the *Dp(1;f)LJ9 *mini-X chromosome. To detect the effect of *CTCF*^*30 *^on the establishment of the imprint, *Dp(1;f)LJ9; e/e *flies were balanced over *X^X; CTCF*^30^/*e *for maternal establishment (Figure 7), or *X^Y; CTCF*^30^/*e *for paternal establishment (Figure 8). *X^X/Dp(1;f)LJ9; CTCF*^30^/*e *females were crossed to *y*^*1*^*z*^*a*^*g*^*53d*^/*Y *males to test maternal imprint establishment (Mat-Est. *CTCF*^*30*^), and the reciprocal cross tested for paternal imprint establishment (Pat-Est. *CTCF*^*30*^). Maternal establishment controls (Mat-Est. *CTCF*^+^) consisted of *X^X/Dp(1;f)LJ9; e/e *females crossed to *y*^*1*^*z*^*a*^*g*^*53d*^/*Y *males, and paternal establishment controls (Pat-Est. *CTCF*^+^) were *X^Y/Dp(1;f)LJ9; e/e *males crossed to *y*^*1*^*z*^*a*^*g*^*53d*^/*y*^*1*^*z*^*a*^*g*^*53d *^females. External controls were also produced by crossing F1 generation *X^X/Dp(1;f)LJ9; e/e *females to *y*^*1*^*z*^*a*^*g*^*53d*^/*Y *males for maternal establishment, and the reciprocal cross for paternal establishment.

To assess the effect of *CTCF*^*30 *^on other variegating strains, *CTCF*^*30 *^/*TM6, Tb *flies were crossed to *In(1)w*^*m4 *^and two variegating fourth chromosome (*6-M193 *or *39C-33*) strains. For the *In(1)w*^*m4*^ crosses, *In(1)w*^*m4 *^females were crossed to *w*^*1118*^/*Y; CTCF*^*30 *^males and the red pigment levels of the *In(1)w*^*m4*^/*Y; CTCF*^*30*^/*+*and *In(1)w*^*m4*^/*w*^1118^*; CTCF*^*30*^/*+ *progeny were compared with that of their *In(1)w*^*m4*^*; TM6, Tb *siblings. Reciprocal crosses were performed with the variegating fourth chromosome strains to control for both the maternal and paternal inheritance of the variegating transgene. The maternal cross consisted of *w-/w-; +/+; +/+; var/var *females crossed to *y/w-; CTCF*^*30 *^/*TM6, Tb; +/+; +/+ *males, and paternal inheritance used *y/w-; +/+; +/+; var/var *males crossed to *w-/w-; CTCF*^*30*^/*+; +/+; +/+ *females, where *var *represents the variegating fourth chromosome transgene. The resulting progeny were separated by sex (*y/w-; CTCF*^*30*^*; +/+; var/var *males and *w-/w-; CTCF*^*30*^/*+; +/+; var/var *females) and compared with the balancer controls (*y/w-; TM6, Tb/+; +/+; var/var *males and *w-/w-; TM6, Tb/+; +/+; var/var *females).

### Measurement of d*CTCF *expression

qRT-PCR was used to measure *dCTCF *expression. Total RNA was prepared from three groups of 50 larvae for each genotype. One microgram of total RNA was reverse transcribed using random hexamers and ImProm-II reverse transcriptase (Promega). Quantitative PCR was performed as previously described [48]. *dCTCF *primers (CTCF F2400, ACGAGGAGGTGTTGGTCAAG and CTCF R2485, ATCATCGTCGTCCTCGAAC) were used at 300 nM. Two technical replicates from each sample were amplified. Expression was normalized to *Dmn*, a gene that has proved reliable for this purpose [48].

### Quantification of eye pigment levels

Expression of the imprint marker gene *garnet *was quantified both visually and through the use a spectrophotometric assay of extracted eye pigments. The visual assay assigns each eye a score in relation to its variegation class as described by Joanis and Lloyd [32]; 0-25% pigmentation, 25-50% pigmentation, 50-75% pigmentation, and 75-100% pigmentation. The prevalence of each variegation class is expressed as a percentage of all eyes assayed. As the variegated phenotype of maternally inherited *Dp(1;f)LJ9 *in the presence of mutant *dCTCF *is skewed toward fully pigmented eyes, a second assay with the following variegation classes was performed: >80% pigmentation, 80-90% pigmentation, 90-100% pigmentation, and 100% pigmentation.

The spectrophotometric assay was adapted from Real *et al*. [49]. For red (pteridine) pigment, flies of each test genotype were aged for 4 days and then placed in 1.5-ml Eppendorf microtubes and stored at -30°C. For each sample set, eight heads were placed into a 0.6-ml microtube containing 400 μl of acidified ethanol (30% EtOH, acidified to pH 2 with HCl). Pigment was extracted on an orbital shaker at 150 rpm in the dark for 48 hours. Absorbance of the extracted pigments was measured at 480 nm. Each 400-μl sample of extracted pigment was split into two 200-μl volumes, independently measured, and the values were averaged. Five tubes were run per sample set, with the values averaged and expressed as a percentage of wild-type pigment levels ± standard deviation. For brown (ommochrome) pigment, flies of each test genotype were aged for 4 days and then placed in 1.5-ml Eppendorf microtubes and stored at -80°C. Ten heads were placed in a 1.5-ml Eppendorf tube and homogenized with 150 μl of 2 M HCl and 0.66% sodium metabisulfite (wt/vol). A total of 200 μl of 1-butanol was added, and the mixture was placed on an orbital shaker at 150 rpm for 30 min before being centrifuged at 9000 *g *for 5 min. The organic layer was removed, washed with 150 μl of 0.66% sodium metabisulfite in dH_2_O and placed back on the orbital shaker for a further 30 min, and then this step was repeated for a second wash. The organic layer was removed and measured for absorbance at 492 nm. Five tubes were run per sample set, with the values averaged and expressed as a percentage of wild-type (O. R) pigment levels ± standard deviation. Absorbance was determined with a Pharmacia Biotech Ultrospec 2000 spectrophotometer. Representative eye pictures were photographed with a Zeiss AxioCam MRc5 mounted on a Zeiss Stemi 2000-C dissecting microscope.

Spectrophotometric assay for quantifying the expression of the *white *transgene on the fourth chromosome variegating strains and *In(1)w*^*m4 *^followed the same procedure for fly aging and head collection. Heads were split into groups of five and placed into 150 μl of 30% EtOH acidified to pH 2 with HCl. Pigment extraction consisted of sonicating samples for 5 seconds at 50 MHz (Sonic 300 Dismembrator Sonicator) prior to soaking samples at room temperature for 24 hours in the dark. For each sample, 90 μl of extracted pigment was loaded into 96-well microtiter trays and quantified with a Microplate Reader (Benchmark Bio-Rad) at a wavelength of 480 nm. The results from each sample group were pooled for a final mean pigment value. Pigment levels for imprint establishment were quantified using the same spectrophotometric assay used for quantifying the *white *variegating strains, except one head was used per sample set.

### Statistical Analysis

Kolmogorov-Smirnov two-sample tests were used to determine the statistical significance between the visual eye scores from control and *dCTCF *mutant data sets. Statistical significance for both red and brown pigments was determined by using an ANOVA followed by Student's *t*-test with Bonferroni-corrected *P *values between the mean of the experimental *dCTCF *mutant data sets and the mean of the results from the appropriate control cross.

### CTCF modENCODE data

The modENCODE data for dCTCF insulator sites from 0- to 12-hour embryos were obtained from the White Lab project on the modENCODE web site http://www.modencode.org[50].

## Authors' contributions

WAM performed the imprinting experiments, provided analysis, and wrote the manuscript. DM characterized the CTCF alleles, NB performed the variegating 4^th ^chromosome strain crosses and assays, GES performed the imprint establishment crosses and assays and VR produced the CTCF^*30 *^allele. VKL and VM participated in the conceptualization and design of the experiments, performed analysis, and participated in writing the manuscript. All authors read and approved the final manuscript.
